# 

**Published:** 1911-08

**Authors:** 


### CONTENTS.

ORIGINAL COMMUNICATIONS—

Surgical Treatment of Typhoid Fever. S. T. Barnett, M. D., Atlanta, Ga. ...... 225

The Diagnosis of Gastric and Duodenal Ulcers. J. Norment Baker, B. A., M. D., Montgomery, Ala. .... 230

Acute Surgical Conditions of the Abdomen Often Mistaken for Appendicitis. By J. L. Campbell, M. D. .... 239

History of Medicine, a Neglected Subject. By Roy Blosser, M. D. .... 244

Therapy of Pulmonary Tuberculosis. By Dr. Louis C. Rouglin, Atlanta, Ga. .. 246

Acute Anterior Poliomyelitis. By T. Jos. Dean, M. D. .... 251

Etiology, Pathology and Treatment of Pellagra; Cotton Seed Oil and Animals. By George C. Mizell, M. D., Atlanta, Ga. .... 260

EDITORIALS .... 278

NEWS AND NOTES .... 279

BOOK REVIEWS .... 281

